# EZH2 variants differentially regulate polycomb repressive complex 2 in histone methylation and cell differentiation

**DOI:** 10.1186/s13072-018-0242-9

**Published:** 2018-12-06

**Authors:** Weipeng Mu, Joshua Starmer, Della Yee, Terry Magnuson

**Affiliations:** Department of Genetics, and Lineberger Comprehensive Cancer Center, The University of North Carolina at Chapel Hill, Chapel Hill, NC 27599-7264 USA

**Keywords:** Polycomb-group proteins, EZH2, Spermatogenesis, Alternative splicing, Histone methylation, Cell differentiation

## Abstract

**Background:**

Polycomb repressive complex 2 (PRC2) is responsible for establishing and maintaining histone H3K27 methylation during cell differentiation and proliferation. H3K27 can be mono-, di-, or trimethylated, resulting in differential gene regulation. However, it remains unknown how PRC2 specifies the degree and biological effects of H3K27 methylation within a given cellular context. One way to determine PRC2 specificity may be through alternative splicing of *Ezh2*, PRC2’s catalytic subunit, during cell differentiation and tissue maturation.

**Results:**

We fully characterized the alternative splicing of *Ezh2* in somatic cells and male germ cells and found that *Ezh*’s exon 14 was differentially regulated during mitosis and meiosis. The *Ezh2* isoform containing exon 14 (ex14-*Ezh2*) is upregulated during cell cycle progression, consistent with a role in maintaining H3K27 methylation during chromatin replication. In contrast, the isoform lacking exon 14 (ex14D-*Ezh2*) was almost exclusively present in spermatocytes when new H3K27me2 is established during meiotic differentiation. Moreover, *Ezh2*’s transcript is normally controlled by E2F transcription activators, but in spermatocytes, *Ezh2*’s transcription is controlled by the meiotic regulator MYBL1. Compared to ex14-EZH2, ex14D-EZH2 has a diminished efficiency for catalyzing H3K27me3 and promotes embryonic stem cell differentiation.

**Conclusions:**

*Ezh2*’s expression is regulated at transcriptional and post-transcriptional levels in a cellular context-dependent manner. EZH2 variants determine functional specificity of PRC2 in histone methylation during cell proliferation and differentiation.

**Electronic supplementary material:**

The online version of this article (10.1186/s13072-018-0242-9) contains supplementary material, which is available to authorized users.

## Background

Polycomb repressive complex 2 (PRC2) catalyzes mono-, di-, and trimethylation of histone H3 lysine 27 (H3K27) and functions as a key epigenetic regulator for normal development and diseases. The three forms of H3K27 methylation are mutually exclusive and form spatially defined genomic domains. Monomethylated H3K27 (H3K27me1) is enriched in the gene bodies of active genes, indicative of its involvement in gene activation [1–4]. In contrast, dimethylated H3K27 (H3K27me2) primarily resides within large intergenic and intragenic chromatin domains (50–70% of H3 tails) [5, 6], possibly exerting a protective role by preventing non-cell-type-specific enhancer use [3]. Lastly, trimethylated H3K27 (H3K27me3) is widely present in the promoter regions of silenced genes and thought to provide PRC2 with a role in transcriptional repression [7, 8]. PRC2 catalyzes H3K27me1 and H3K27me2 relatively efficiently compared to H3K27me3 [9]. During cell cycle progression, H3K27me1 and me2 are rapidly incorporated into chromatin after DNA synthesis, but the restoration of normal H3K27me3 levels is delayed [10–12]. In light of PRC2’s versatility in histone methylation and gene regulation, it is important to understand how this complex is spatiotemporally orchestrated to load the appropriate methyl groups onto targeted genomic loci during cell proliferation and differentiation.

The PRC2 complex is composed of core subunits (EED, EZH1/2, SUZ12, and RBBP4/7) required for enzymatic activity, and accessory subunits (PCLs, JARID2, AEBP2) that regulate the recruitment and activity of PRC2 [7]. Recent studies suggest that accessory PRC2 components regulate DNA binding, methyltransferase activity and the spread of methylation marks on the genome. The selective incorporation of accessory subunits leads to the formation of PRC2 subcomplexes, PRC2.1, characterized by the incorporation of AEBP2 and JARID2, and PRC2.2, characterized by mutually exclusive binding of one of the three polycomb-like homologs (PCLs) PHF1, PHF19, and MTF2 [13]. However, these PRC2 variants only exhibit subtle differences in histone methylation and do not explain the locus-specific H3K27 methylation across the whole genome. In contrast, with the exception of EED, little is known about the roles that the core subunits may play in determining PRC2’s flexibility in histone methylation. EED undergoes alternative translation to yield four protein products; however, this diversity does not control the number of methyl groups added to H3K27 since each isoform is sufficient to generate all three H3K27 methylation marks [14].

PRC2 plays two major roles in histone methylation: it can maintain existing H3K27 methylation marks so that they are propagated to daughter cells and it can establish new H3K27 methylation sites. During cell proliferation, the E2F family of cell cycle transcription factors enhances the expression of *Ezh2,* which duplicates H3K27 methylation during cell proliferation [15]. During cell differentiation, PRC2 establishes new H3K27 methylation sites, especially in male germ cells. These new H3K27 methylation marks are introduced into the genome to determine the cell-type-specific transcriptome [16]. In this case, PRC2’s recruitment to specific loci for methylation appears to be a more complicated process, potentially involving noncoding RNAs, sequence-specific transcription factors, and/or PRC2-interacting proteins with affinity for CpG-rich DNA elements [17]. Our previous results demonstrate that EED, EZH2, and SUZ12 are dramatically upregulated in pachytene spermatocytes [16], suggesting the germ cell-specific PRC2 complex has a role in establishing H3K27 methylation during meiotic progression. Therefore, it is crucial to understand how PRC2 can distinguish these two different types of histone methylation coupled with cell proliferation and differentiation.

EZH2 may have a major role in determining PRC2’s differential methylation roles. It possesses multiple interaction domains for EED and SUZ12, facilitating the methyltransferase activity conveyed by its SET domain [18–20]. EZH1, a homolog of EZH2 encoded by a separate locus [21], has much less methyltransferase activity and cannot substitute for EZH2 in histone methylation and related biological functions in many tissues [22]. Because other PRC2 subunits only have a subtle effect on EZH2 methyltransferase specificity, we speculate that EZH2’s variants themselves diversify PRC2’s functional roles in distinct methylation processes during cell proliferation and differentiation.

Here, we identified multiple *Ezh2* isoforms derived from alternative transcriptional splicing in various tissues and cell types. Expressions of EZH2 variants that include or exclude exon 14 are differentially regulated via cell cycle or meiotic regulators, respectively, during mitosis and meiosis. The EZH2 isoform without exon 14 (ex14D-EZH2*)* has a disrupted CXC domain and is the primary isoform found in spermatocytes. This isoform is responsible for the establishment of H3K27me2, but is less efficient at catalyzing H3K27me3. Moreover, exclusive expression of ex14D-EZH2 in ES cells promotes their differentiation, indicated by precocious and enhanced expression of mesoderm genes. In contrast, the EZH2 isoform with exon 14 (ex14-EZH2) is the most common isoform in proliferating cells and more efficient at catalyzing H3K27me3. Our study suggests that the incorporation of specific EZH2 variants into the PRC2 complex controls the appropriate level and extent of H3K27 methylation in polycomb target loci during the establishment and maintenance of these epigenetic marks.

## Results

### *Ezh2* pre-mRNA splicing is differentially regulated during meiosis and mitosis

**Fig. 1:**
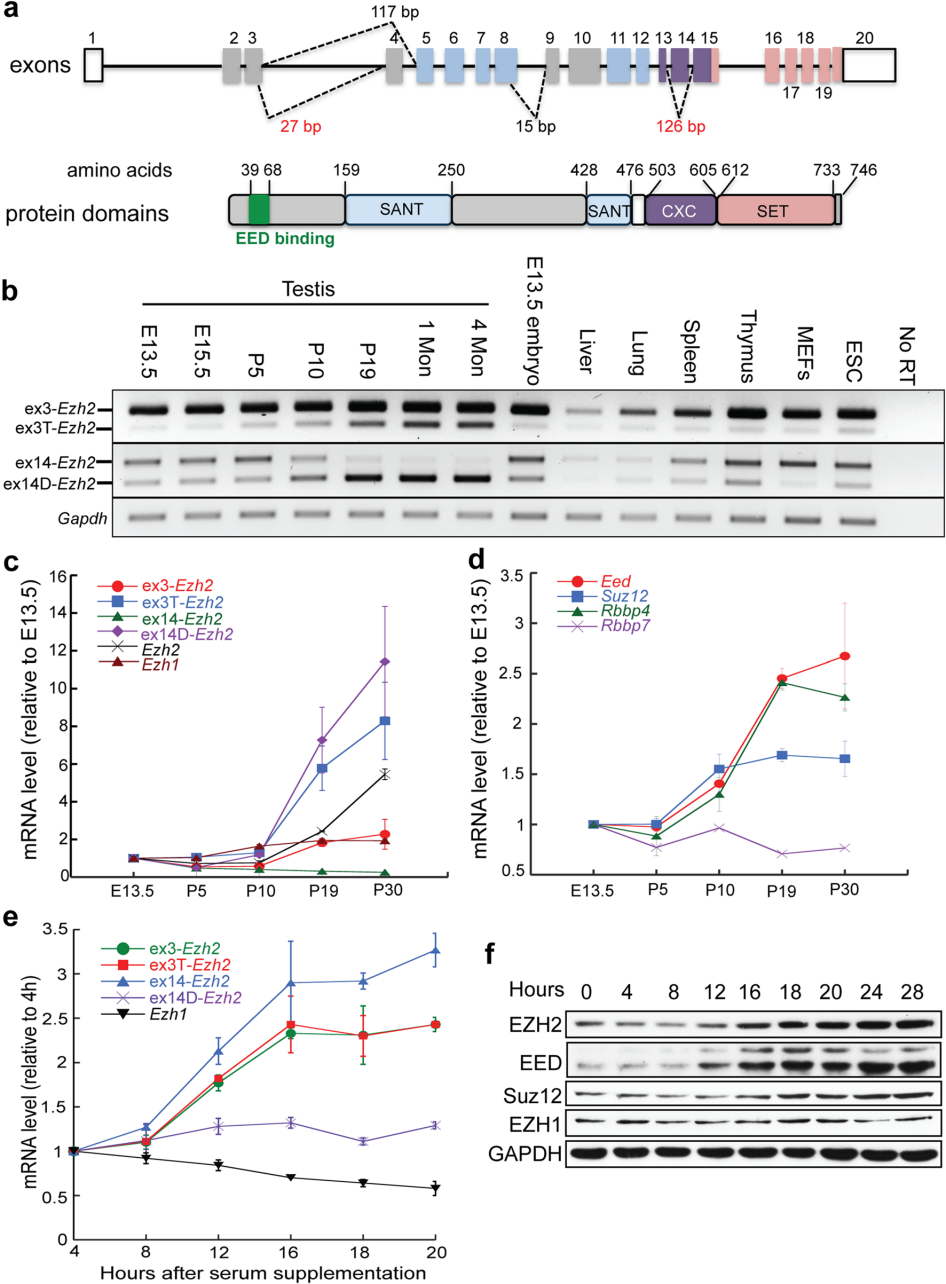
*Ezh2* splicing is differentially regulated during meiosis and mitosis. **a** Schematic structure of the mouse *Ezh2* gene and protein. Removal of exon 14 causes the disruption of the CXC domain. **b** RT-PCR analysis of alternative *Ezh2* transcripts in mouse testis at different ages, tissues, embryos, and cell lines. **c** Quantitation of *Ezh1* and *Ezh2* transcripts during testis development by qPCR analysis. **d** Quantitation of the transcription of PRC2 core components by qPCR analysis. **e** Quantitation of *Ezh1* and *Ezh2* transcripts during the cell cycle progression. **f** Western blot analysis of PRC2 core components during the cell cycle progression

*Ezh2* makes several distinct transcripts due to alternative splicing. Exons 4 and 14 can be skipped and exons 3 and 8 can be truncated (Fig. 1a) [23]. To determine whether different *Ezh2* transcripts are cell and tissue type specific, we profiled *Ezh2* transcripts in different ages of testes, somatic tissues, embryos, and primary cell lines by RT-PCR. The *Ezh2* transcripts that contain alternative splicing for exon 3 and exon 14 are found in many tissues and cultured cells (Fig. 1b). In contrast, transcripts containing alternative splicing for exons 4 and 8 were barely detected (Additional file 1: Fig. S1). Thus, we focused on *Ezh2* transcripts with variations in exons 3 and 14.

First, we examined *Ezh2* and *Ezh1* transcript levels during spermatogenesis. *Ezh2* transcripts without exon 14 (ex14D-*Ezh2*) were at a relatively low level in mitotic germ cell populations such as progenitors (E13.5 and E15.5) and spermatogonial stem cells (p5). However, this isoform was dramatically upregulated at the time of entry into meiosis (p10) and maintained at high levels during the meiotic progression as shown in p19, 1-month and 4-month testes. In contrast, the *Ezh2* transcripts containing exon 14 (ex14-*Ezh2*) were downregulated during meiosis (Fig. 1b). The dynamics of exon 14-related alternative transcripts were verified by qPCR analysis (Fig. 1c). Transcription of PRC2’s core components, *Eed*, *Suz12*, and *Rbbp4*, also increased during germ cell maturation (Fig. 1d), suggesting ex14D-EZH2 functions within the PRC2 complex during meiotic differentiation. In contrast, *Ezh1* levels were consistent throughout germ cell development (Fig. 1c), indicating its expression is independent of meiotic differentiation.

Because ex14-*Ezh2* is abundant in mitotic germ cells and rapidly dividing ES cells and primary MEFs (Fig. 1b), we wanted to determine the dynamics of *Ezh2* and *Ezh1* transcripts during mitosis. Thus, we synchronized primary MEFs at the G0/G1 phase by serum starvation and then released them into the S and G2/M phases with serum supplementation. In comparison with meiosis, ex14-*Ezh2*, but not ex14D-*Ezh2*, was remarkably elevated during mitotic progression (Fig. 1e). The *Ezh2* transcripts with a full exon 3 (ex3-*Ezh2*) or a truncated exon 3 (ex3T-*Ezh2*) were both upregulated. EZH2, EED, and SUZ12 protein levels also increased in mitotic cells (Fig. 1f). *Ezh1* expression decreased with the cell cycle activation, which is consistent with the high expression of *Ezh1* in mature tissue but low in proliferating tissues [21]. These results indicate that *Ezh* variants are differentially regulated during meiosis and mitosis.

### ex14D-*Ezh2* transcription is independent of E2F regulation and responsible for establishing H3K27me2 in spermatocytes

**Fig. 2:**
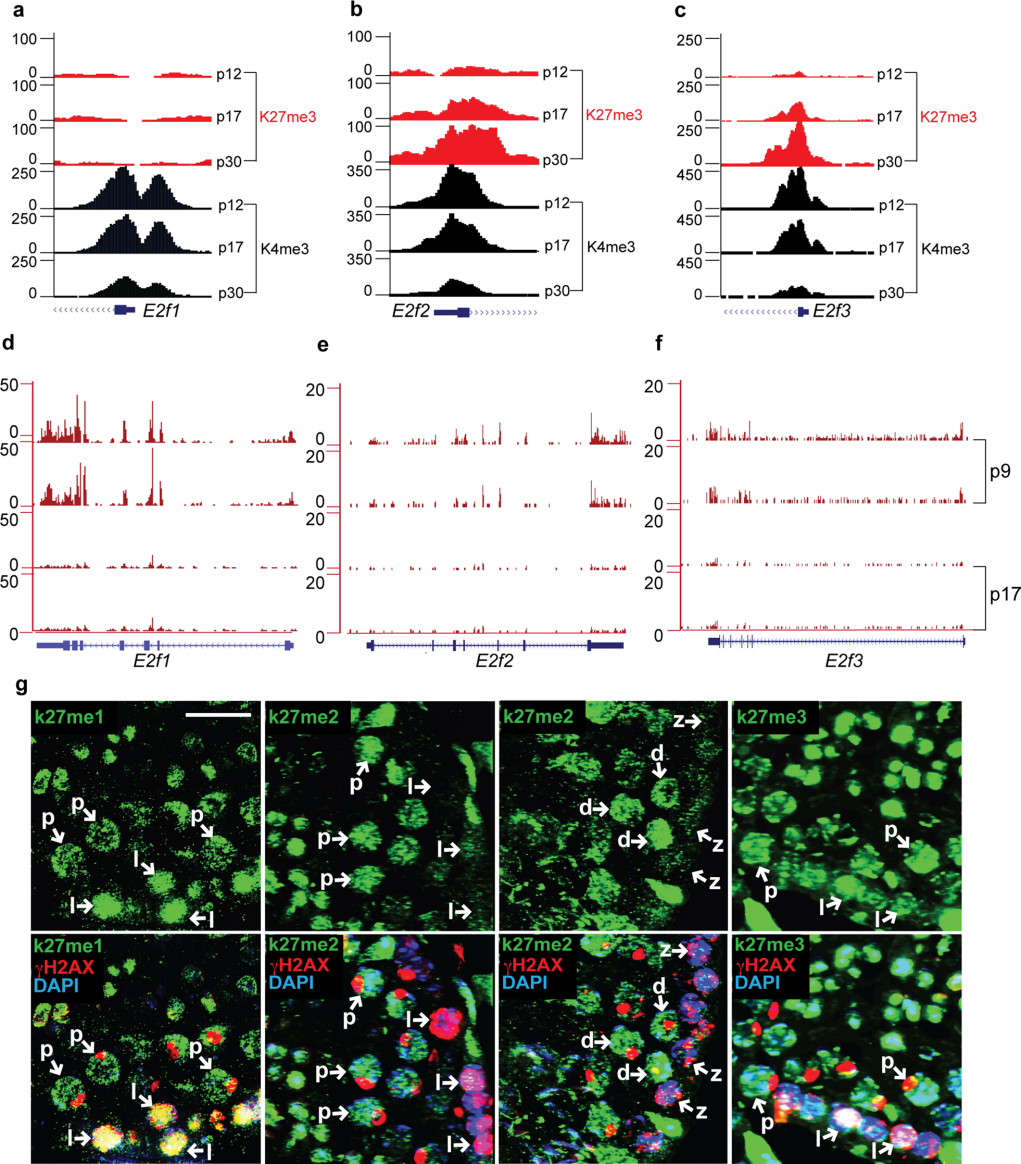
The expression of ex14D-*Ezh2* in spermatocytes is responsible for the establishment of H3K27me2. **a**–**c** The dynamics of H3K27me3 and H3K4me3 enrichment on *E2f1*-*3* during spermatogenesis. Spermatocytes were isolated from postnatal day 12 (p12), 17 (p17), and 30 (p30) testes for ChIP-seq analysis. **d**–**f** RNA-seq analysis of *E2f1*-*3* transcription in germ cells purified from p9 and p17 testes. The representative sample tracks for each time point are presented here. **g** Assessment of H3K27 methylation in different meiotic cell populations. Cryosections of 4-month-old testis were immunostained for indicated proteins. (l) leptotene spermatocytes; (z) zygonema; (p) pachynema; (d) diplonema. Scale bar: 20 μm

*Ezh2* expression is typically regulated by the E2F family of transcription factors, among which E2F1-3 function as activators to promote *Ezh2* transcription in proliferating cells [15]. To assess transcriptional reprogramming of *E2f1*-*3* during spermatogenesis, we analyzed their chromatin state and expression levels in germ cells isolated from different ages of testes. We found repressive histone marks, H3K27me3, accumulated around *E2f2* and *E2f3*’s transcriptional start sites in germ cells over time, with the most H3K27me3 detected at p30. This was the opposite of the accumulation of active marks, H3K4me3 (Fig. 2a–c). Accordingly, *E2f1*-*3* transcription was active in p9 testes with ample spermatogonial populations but repressed in meiocyte-enriched p17 testes (Fig. 2d–f), suggesting that E2F does not contribute to upregulated *Ezh2* expression during meiotic progression.

Meiotic ex14D-*Ezh2* expression occurs at a time when new histone marks are established during cell differentiation, suggesting that it may have a role in this process. Thus, we characterized H3K27 methylation patterns in different populations of spermatocytes during meiosis. There were no significant differences in H3K27me1 and H3K27me3 levels between early stage (leptonema) and late stage (pachynema) spermatocytes. However, H3K27me2 levels were strikingly elevated during the zygonema to pachynema transition (Fig. 2g), which is consistent with the upregulation of EZH2 in pachytene spermatocytes [16] and with ex14D-*Ezh2* transcripts in pachynema-enriched p19 testes (Fig. 1b, c). These results suggest that ex14D-*Ezh2* transcription and processing occur in a cellular context-dependent manner for establishing H3K27me2.

### MYBL1 activates ex14D-*Ezh2* expression in spermatocytes

**Fig. 3:**
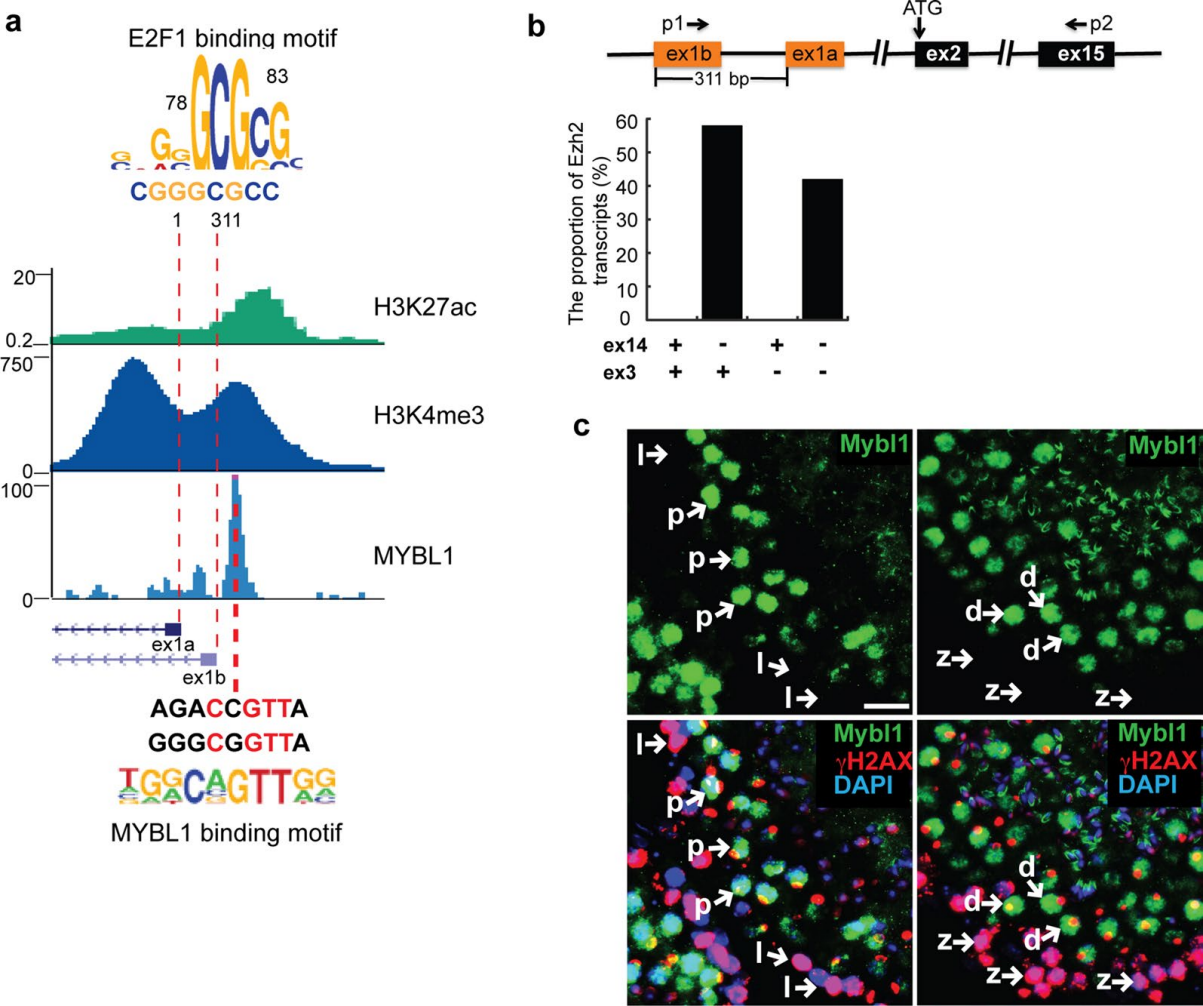
The expression of *ex14D*-*Ezh2* is activated by MYBL1. **a** The epigenetic signature and transcription factor binding motifs around *Ezh2*’s 5′ UTR. HOMER-motif analysis detected E2F1 binding sites within the 300 bp upstream of the ex1a TSS. ex1a is the first exon that is known for coding *ex14*-*Ezh2*’s 5′ UTR. ex1b is a putative exon for ex14D-*Ezh2*’s 5′ UTR. **b** Characterization of *ex14D*-*Ezh2*’s 5′ UTR. Primer p1 and p2 were used to amplify the DNA sequence coding the transcripts initiated from the ex1b 5′ UTR. The amplicons were cloned into vectors and transformed into bacteria. The alternative splicing events were assessed by genotyping single colonies. **c** Cryosections of p30 testes were immunostained with indicated proteins. Scale bar: 20 μm

Because ex14D-*Ezh2* expression was increased during meiosis, we speculated that it was regulated by meiosis-specific transcriptional activators. We did not observe any active chromatin marks, such as H3K4me3, H3K27ac, or ATAC-seq peaks, throughout the ENSEMBL-annotated transcript for ex14D-*Ezh2* (Additional file 2: Fig. S2a), suggesting that transcription of this isoform initiated from outside of the transcript. We predicted that the other two ENSEMBL-annotated noncoding exons, ex1a and ex1b, which are surrounded by H3K4me3 and H3K27ac in testis, could encode ex14D-*Ezh2*’s 5′ UTR (Fig. 3a). The first noncoding exon, ex1a, contains an E2F1 binding motif, as predicted by HOMER (Fig. 3a), that is bound by E2F1 as determined by a ChIP-seq analysis, in proliferating human Hela S3 cells (Additional file 2: Fig. S2b) (ENCODE/Peggy Farnham). This suggests the existence of a promoter in that region drives the cell cycle-related expression of ex14-*Ezh2* and not ex14D-*Ezh2*, since ex14D-*Ezh2* transcription is not promoted by E2F1. Thus, we suspected that the second noncoding exon, ex1b, encoded the 5′ UTR of ex14D-*Ezh2* in testis. To test this, we performed RT-PCR analysis to amplify cDNA fragments spanning exon 1b to exon 15, and then cloned the amplicons into plasmids for genotyping the alternative splicing at exon 3 and ex14 by Colony PCR assays (Fig. 3b). We found that the amplicons derived from ex1b excluded exon 14, demonstrating ex1b encodes the 5′ UTR of ex14D-*Ezh2*. ex14D-*Ezh2* did not have preference for the alternative splicing of exon 3.

Because ex1b encodes the 5′ UTR of ex14D-*Ezh2*, we performed motif analysis with HOMER to search for transcription factors that could bind near it. This analysis predicted that MYBL1, a master regulator of male meiosis [24], had a DNA binding site upstream of ex1b, and binding was confirmed by MYBL1 ChIP-seq analysis (Fig. 3a) [25]. Consistent with *Ezh2* expression in meiosis, MYBL1 was at a low level in leptotene and zygotene, and significantly elevated in pachytene and diplotene spermatocytes (Fig. 3c). MYBL1’s role as a transcriptional activator of *Ezh2* in germ cells is further supported by the evidence that a *Mybl1* knockout in testis caused the downregulation of *Ezh2* transcription [24]. MYBL1 was also enriched in the promoter regions of *Eed* and *Suz12* (Additional file 2: Fig. S2c, d), indicative of its functioning as a PRC2 regulator during spermatogenesis. Thus, all of these data suggest that MYBL1 activates ex14D-*Ezh2* expression from ex1b in spermatocytes.

### ex14D-EZH2 has preferential activity toward H3K27me2 but not H3K27me3 and promotes ES cell differentiation

**Fig. 4:**
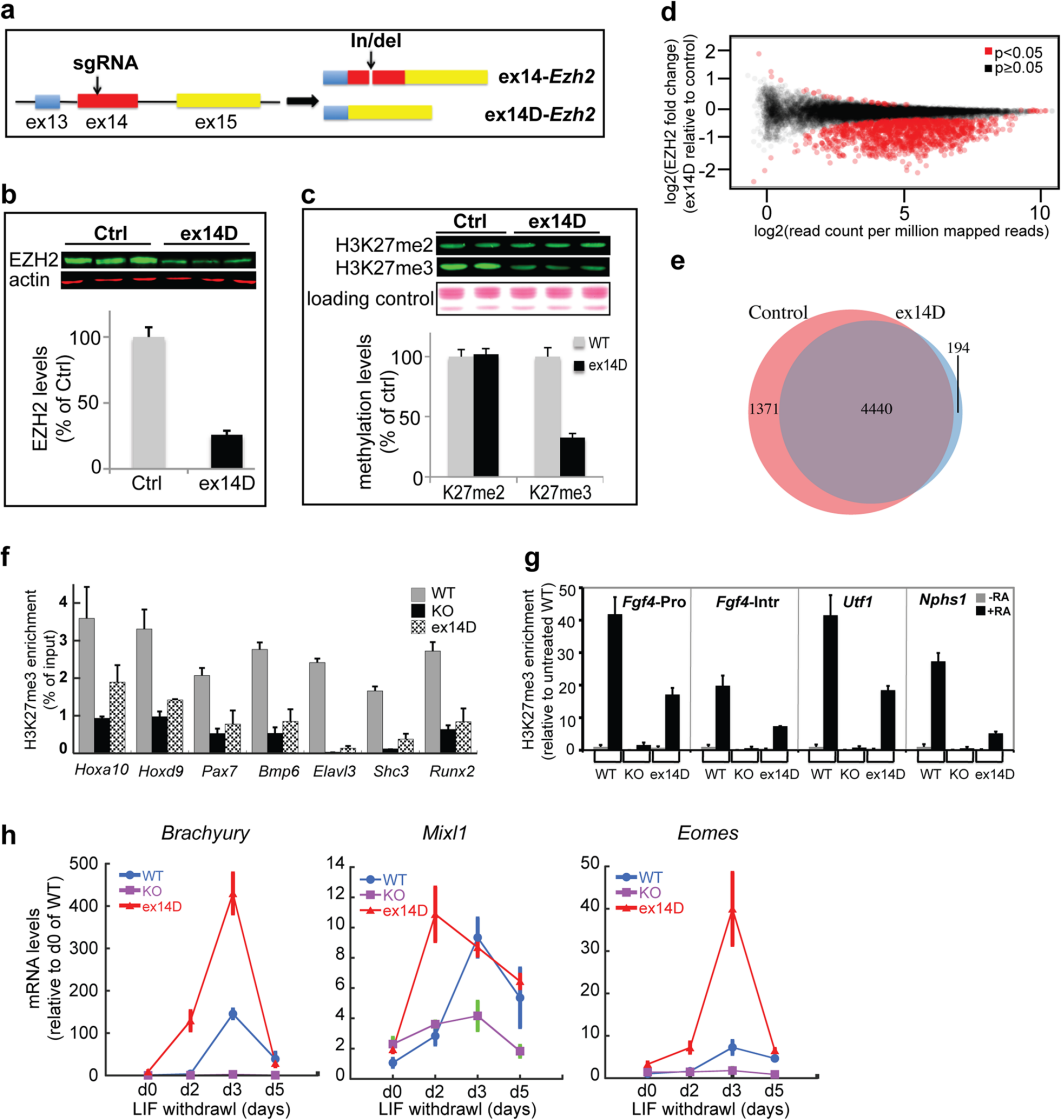
ex14D-EZH2 participates in establishing and maintaining H3K27 methylation and promotes ES cell differentiation. **a** A schematic of how ex14D-*Ezh2* ES cell lines were created by CRISPR-Cas9. **b** Western blot analysis of EZH2 proteins in ex14D-*Ezh2* cells. **c** Western blot analysis of histone H3K27 methylation levels in ex14D-*Ezh2* ES cells. **d** A smear plot showing ex14D-EZH2 is not sufficient for saturating EZH2 targeting genes. **e** A Venn diagram showing that ex14D-EZH2 binds to the majority EZH2 targets. **f** Assessment of H3K27me3 maintenance on key developmental genes in ex14D-*Ezh2* ES cells by ChIP-PCR analysis. **g** Assessment of the newly introduced H3K27me3 marks during ES cell differentiation. **h** qPCR analysis of the expression of mesoderm marker genes during LIF withdrawal-induced ES cell differentiation

To explore the roles of ex14D-EZH2 in H3K27 methylation and cell differentiation, we generated ES cell lines in which *Ezh2*’s exon 14 was disrupted using CRISPR-Cas9. The in/del mutation on exon 14 caused a reading frame shift, resulting in the disruption of the SET domain in ex14-EZH2. Thus, only the alternatively spliced ex14D-EZH2 isoform was functional (Fig. 4a). EZH2 protein levels in ex14D-*Ezh2* cell lines were around 30% of those in control cell lines, demonstrating the loss of ex14-EZH2 (Fig. 4b). Consistent with the EZH2 reduction, H3K27me3 levels were decreased by 67% in the mutant cell lines. However, there was no reduction in H3K27me2 levels in ex14D-*Ezh2* ES cells compared to the controls (Fig. 4c). To assess if ex14D-EZH2 could still saturate EZH2 binding sites, we performed ChIP-seq analysis on EZH2 and found that the enrichment of EZH2 was decreased in the target genes in ex14D-*Ezh2* cells (Fig. 4d). Although EZH2 protein was decreased by 70%, ex14D-EZH2 still methylated 76% of the target genes (4440 out of 5811 genes) by H3K27me3 (Fig. 4e). We quantified the enrichment of H3K27me3 in two categories of genes: genes with H3K27me3 marks all over the gene body, including *Hoxa10*, *Hoxd9*, *Bmp6* and *Pax7*, and genes with this mark around TSS region, such as *Elavl3*, *Shc3*, and *Runx2*. Both groups of genes were less methylated when only ex14D-EZH2 was present (Fig. 4f). Thus, ex14D-EZH2 maintains normal levels of H3K27 dimethylation but not H3K27 trimethylation.

PRC2 can establish new H3K27me3 marks for reprogramming the transcriptome during cell lineage specification. De Novo trimethylation of H3K27 occurs during retinol acid-induced ES cell differentiation [26]. In RA-treated ES cells, we found that ex14D-EZH2 introduced new H3K27me3 marks to *Fgf4*’s promoter and intron, *Utf1*, and *Nphs1*, but at reduced levels (Fig. 4g). In contrast, *Ezh2* knockout cells failed to establish those marks during ES cell differentiation, suggesting that EZH1 cannot establish new H3K27me3 marks even though it can partially rescue the maintenance of H3K27me3 during cell proliferation as shown in Fig. 4f.

Because EZH2 is required for mouse ES cell differentiation [27], we tested if ex14D-*Ezh2* had a specific role in this process. Withdrawal of leukemia inhibitory factor (LIF) induces differentiation of mouse ESCs into various embryonic and extraembryonic lineages [28]. To understand to what degree ex14D-EZH2 contributes to ES cell differentiation, we measured the expression of mesoderm marker genes activated by LIF withdrawal in *Ezh2*KO, ex14D-*Ezh2*, and control cells. As expected, in *Ezh2* knockout cells, *Brachyury*, *Eomes*, and *Mixl1* failed to activate. In contrast, *Brachyury*, *Eomes*, and *Mixl1* were all upregulated in ex14D-*Ezh2*, and control cells. Intriguingly, exclusive expression of ex14D-*Ezh2* led to precocious expression of these marker genes at higher levels (Fig. 4h). In conclusion, these data suggest that ex14D-*Ezh2* promotes ES cell differentiation.

### EZH1 and EZH2 variants have differential activity in catalyzing trimethylation of H3K27

**Fig. 5:**
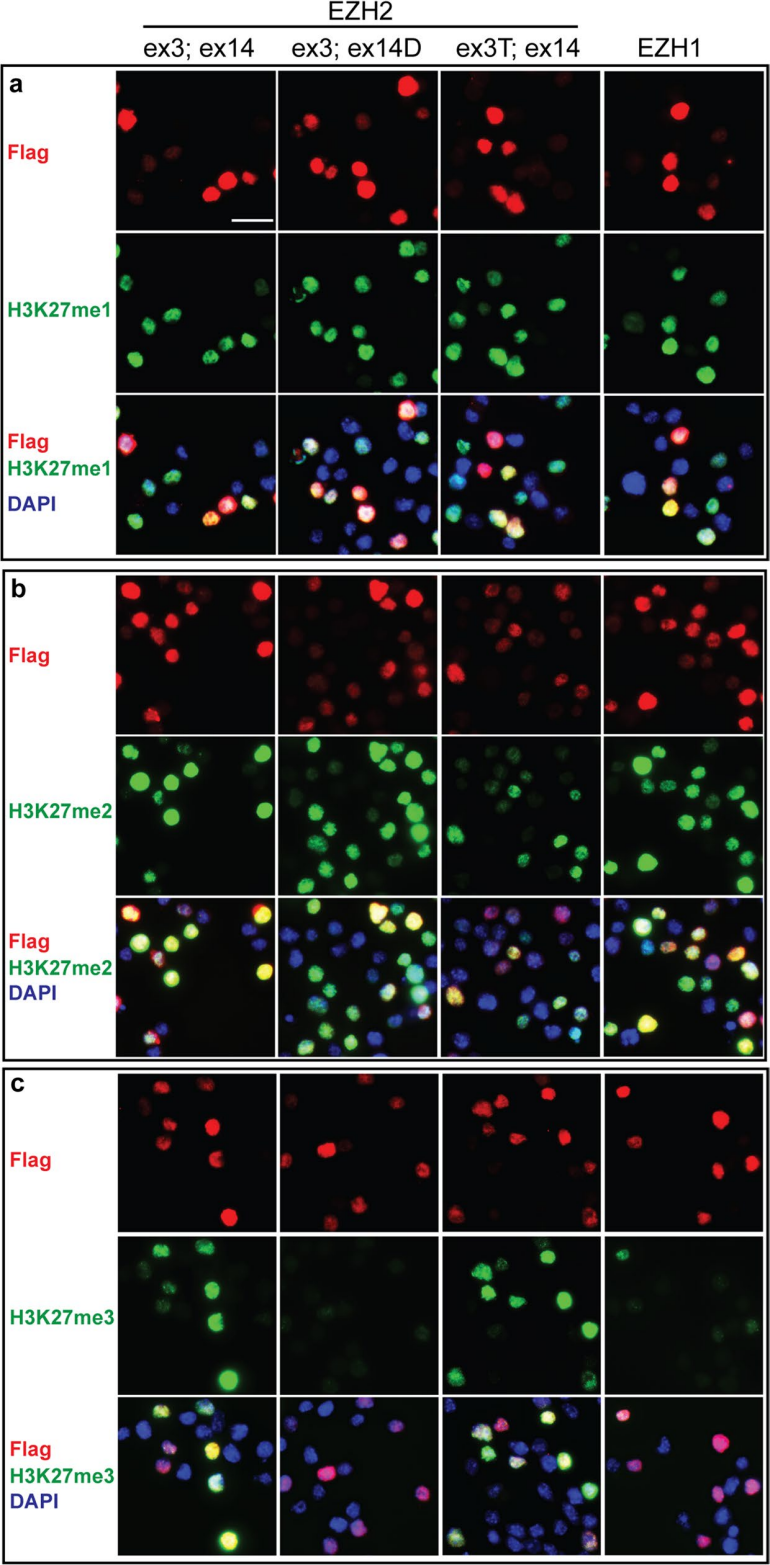
EZH variants had different abilities to catalyze the trimethylation of H3K27. **a**–**c** Immunofluorescence analysis of H3K27me1, me2, and me3 in *Ezh*DKO ES cells that were transfected with FLAG-tagged EZH variants, respectively. Scale bar: 20 μm

The reduction in H3K27me3 in ex 14D-*Ezh2* ES cells (Fig. 4c) could be due to the decreased EZH2 levels (Fig. 4b) and/or the weak methyltransferase activity of ex14D-EZH2 toward H3K27me3. To compare the methyltransferase activity of EZH1 and EZH2 variants, we ectopically expressed *Ezh1* and *Ezh2* isoforms with different combinations of alternative splicing on exon 3 and exon 14 in *Ezh2*- and *Ezh1/2*-deficient ES cells. The *Ezh2*KO caused dramatic decreases in H3K27me2 and H3K27me3, but did not affect H3K27me1 levels. The *Ezh1*/*2* double knockout (*Ezh*DKO) depleted H3K27me2 and H3K27me3 even more than the *Ezh2*KO in addition to reducing H3K27me1 (Additional file 3: Fig. S3a). Because Western blot analysis showed that the EZH2 isoforms, in the absence of exon 14, were expressed at relatively low levels (Additional file 3: Fig. S3b), we used immunofluorescence to assess the H3K27 methylation levels in single cells. We used the same exposure times, contrast and brightness adjustments to ensure EZH2 intensity was comparable among different variants. We found that EZH2 variants containing exon 14 can establish all three forms of H3K27 methylation (Fig. 5 and Additional file 3: Fig. S3c, d). In contrast, the absence of exon 14 severely impaired EZH2’s ability to trimethylate H3K27 (Fig. 5c and Additional file 3: Fig. S3d), but did not affect the mono- and dimethylation (Fig. 5a, b, and Additional file 3: Fig. S3c). *Ezh1* also exhibited weak activity toward tri-, but strong H3K27 mono- and dimethylation activity in ES cells (Fig. 5 and Additional file 3: Fig. S3c, d). Thus, EZH1 and EZH2 variants have differential activity for methylating H3K27.

Since EZH variants have different abilities with respect to H3K27 methylation, we asked if the physical interactions between EZH variants and core subunits or accessory subunits regulate their catalytic specificity and activity. EZH2 in ES cells and ex14D-EZH2 in spermatocytes can interact with SUZ12 and EED (Additional file 4: Fig. S4a). In HEK293T cells cotransfected with EZH variants and PRC2 subunits, we found that PHF1, PHF19, JARID2, and RBBP4 were part of the protein complexes containing either ex14D- or ex14-Ezh2 (Additional file 4: Fig. S4b, c). These results suggest that EZH variants do not selectively exclude these subunits when assembled into PRC2. Taken together, the alteration of EZH2’s CXC domain determines its SET domain capacity in H3K27me3.

## Discussion

For most histone methylation processes, such as H3K9me and H3K4me, multiple methyltransferases participate in loading a specific number of methyl groups onto histones, resulting in gene regulation [29]. However, the three forms of H3K27 methylation are mostly executed through EZH2-PRC2. H3K27me1 and H3K27me2 are found throughout the chromosomes, but without the coexistence of PRC2 by ChIP-seq analysis [3]. In contrast, chromatin domains marked by H3K27me3 are also abundant in PRC2 binding [16]. This suggests that PRC2 uses differential mechanisms to control the methylation reaction on its targeting loci. Although other PRC2 subunits either regulate or are essential for EZH2’s enzymatic activity, they do not determine the level of methylation (i.e., H3K27me1, me2 and me3). EZH1 and EZH2 variants contain multiple domains that mediate physical interactions with most of PRC2’s subunits and ncRNA [30]. We hypothesize that the diversity in EZH1 and EZH2 proteins regulate H3K27 methylation and establish and maintain these epigenetic marks. By examining the dynamics of *Ezh2* transcripts during cell proliferation and differentiation, we showed that EZH2 variants with and without exon 14 mechanistically regulated PRC2’s ability to load specific numbers of methyl groups onto histones.

*Ezh2*’s exon 14 encodes a CXC domain which precedes the SET catalytic domain and is characterized by three C-X(6)-C-X(3)-C-X-C motifs [31, 32]. Mutation of the cysteine in CXC domains impaired the methyltransferase activity in PRC2 [33] and overexpression of the protein interacting with a CXC domain in EZH2 can enhance H3K27 methylation [34]. Consistent with these two studies, ex14D-EZH2, which lacks two C-X(6)-C-X(3)-C-X-C motifs in the CXC domain, exhibited diminished catalytic activity in H3K27me3. But this disruption of the CXC domain did not affect PRC2’s ability to mono- and di-methylate H3K27. We also found that nearly exclusive expression of ex14D-*Ezh2* is coincident to acquiring H3K27me2 in spermatocytes. This led us to speculate that *Ezh2*’s alternative splicing serves as a switch to control the methylation status on modified nucleosomes. Because EZH2 can convert H3K27me2 to H3K27me3 [35, 36], ex14D-EZH2 in spermatocytes may help to maintain the H3K27me2 marks which fail to be trimethylated at genomic loci where silencing normally involves H3K27me3-independent mechanisms. Previous studies showed that the H3K27me3 marks accumulated on key developmental genes were not replaced by protamines in sperm [37, 38] and could be transmitted to the next generation for regulating embryonic gene expression [39]. Thus, it is important to maintain the genomic distribution of H3K27 methylation states in germ cells for epigenetic inheritance.

EZH1 was thought to compensate for the absence of EZH2 in H3K27 methylation. But in our study, EZH1 was unable to generate new H3K27me3 marks on target genes during cell differentiation. Also, overexpression of EZH1 did not rescue H3K27me3 in proliferating cells as ex14-EZH2 did, even though EZH1 and EZH2 are nearly identical in their CXC and SET domains. However, compared to EZH2, EZH1 does not have a cell cycle-regulated expression pattern, it shares less similarity in the N-terminal domains, and it promotes mRNA transcription during cellular differentiation [40]. This indicates that the methylation activity of EZH1-PRC2 is regulated differently from EZH2-PRC2. In contrast to EZH2, EZH1 is abundant in highly differentiated tissues and low in actively proliferating cells. It will be interesting to explore EZH1’s cell-type-specific interacting partners, which facilitate the recruitment onto chromatin and the activation of its methyltransferase activity.

EZH2 level is a determinant for its appropriate biological functions. Deficiency in EZH2 caused cell cycle arrest, senescence, and differentiation defects [15]. EZH2 overexpression has been reported to promote cell proliferation and neoplastic transformation [41]. Our findings show that EZH2 expression is finely controlled at both transcriptional and post-transcriptional levels for producing suitable doses and isoforms of EZH2. To regulate transcription, cell-type-specific TFs and cell cycle-related TFs cooperate to adjust EZH2 expression levels according to the cell’s differentiation and proliferation status. The levels of ex14D-EZH2 for generating new H3K27me in spermatocytes are much lower than EZH2 for duplicating H3K27 methylation in highly proliferating ES cells (Additional file 4: Fig. S4d). The relatively low levels of ex14D-EZH2 in differentiating cells could serve as a mechanism for recovery of H3K27 methylation due to histone turnover occurring at a low rate [42] and, meanwhile, introducing new marks at specific genomic loci. For pre-mRNA processing, each alternative splicing event is controlled by multiple RNA binding proteins (RBPs), the combined action of which creates a distribution of alternatively spliced products in a given cell type [43]. In renal cancer cells, the splicing factor SF3B3 stimulates the inclusion of exon 14 in *Ezh2* transcripts [44]. During spermatogenesis, the splicing landscape is globally reprogrammed in the mitotic-to-meiotic transition during the germ cell cycle [45]. We reason that spermatocytes express a meiosis-specific array of RBPs, which give rise to the skewed production of ex14D-*Ezh2* transcripts.

**Fig. 6:**
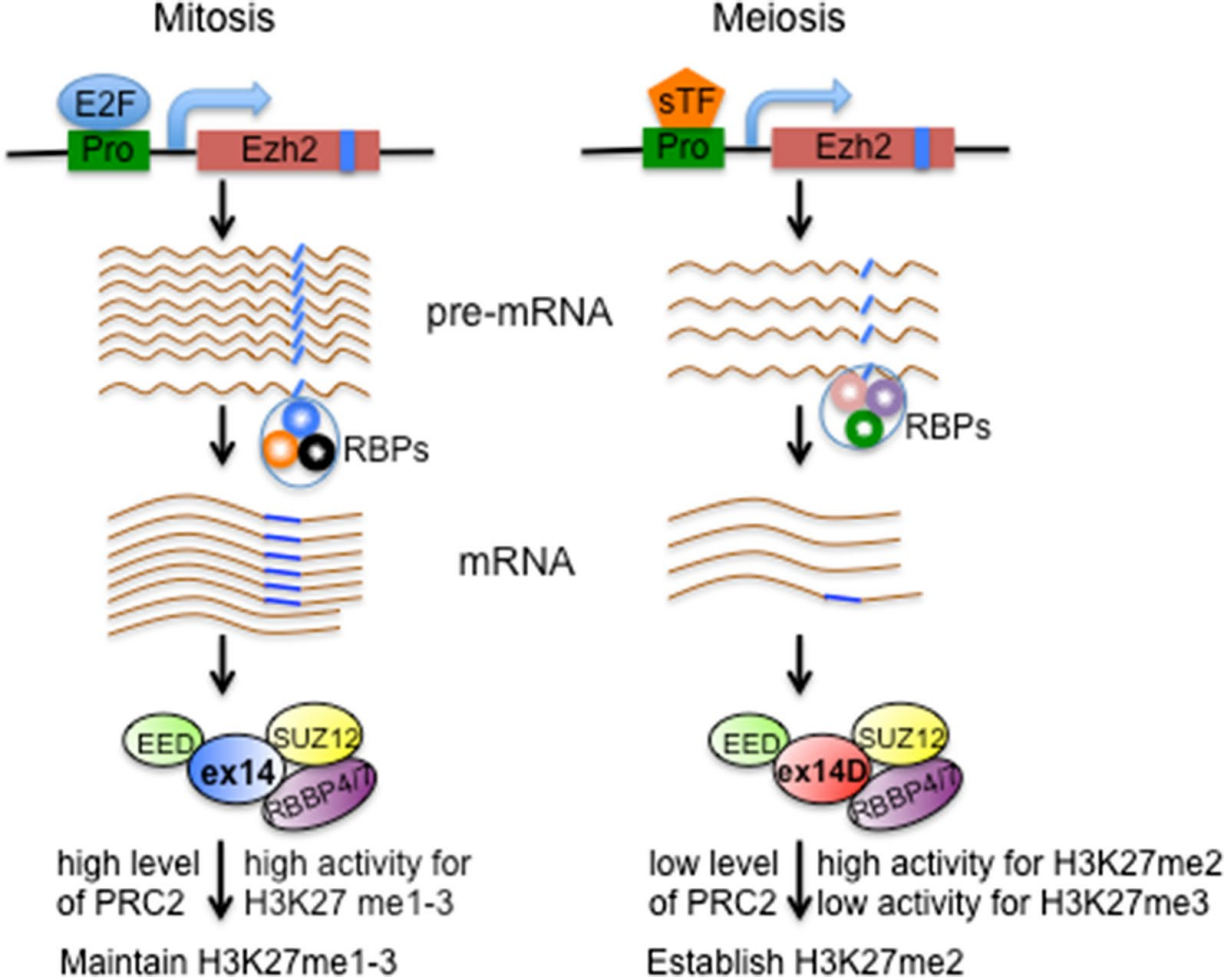
A model for *Ezh2* variant expression and regulation of PRC2 in histone methylation. *Ezh2* transcriptional levels are controlled by E2F and a cell-type-specific transcriptional factor (sTF) to fulfill the required histone methylation. The ratio of EZH2 variants is regulated through distinct combination of RNA binding proteins (RBPs), which determines the fidelity and efficiency for establishing and maintaining H3K27 methylation. The blue rectangles and lines indicate exon 14 of *Ezh2*

We propose a model for regulating EZH2 variants to establish and maintain H3K27 methylation (Fig. 6). During mitosis, the E2F cell cycle regulators stimulate ex14-EZH2-PRC2 expression at high levels to duplicate H3K27 methylation marks across the genome in progeny cells. During meiosis, germ cell-specific transcription factors and splicing factors control exclusive expression of ex14D-EZH2. The low levels of ex14D-EZH2-PRC2 are active for dimethylation of H3K27, but with decreased capability for H3K27me3, resulting in the faithful establishment of new H3K27me2 marks.

## Conclusions

We revealed that *Ezh2*’s expression was differentially regulated at transcriptional and post-transcriptional levels during mitosis and meiosis. EZH2 isoforms have different catalytic activity in mono-, di-, and trimethylation of H3K27. The incorporation of specific EZH2 variants into the PRC2 complex regulates the fidelity in the establishment and maintenance of these epigenetic marks across the genome.

## Materials and methods

### RT-PCR and qPCR

Total RNA was extracted from whole testis or isolated spermatocytes using TRIzol reagent (Invitrogen) followed by an RNA cleanup step and on-column DNA digestion using Direct-zol™ RNA MiniPrep Kit (Zymo Research) according to the manufacturers’ instructions. cDNA was synthesized using ProtoScript II Reverse Transcriptase (NEB). Real-time qPCR analysis was performed using SsoFast EvaGreen supermix (Bio-Rad) and CFX96 thermocycler (Bio-Rad) (Additional file 5: Table S1).

### ChIP-seq

The ChIP-seq was performed as described [46]. Five million mouse ES cells and 2.5 μg of H3K27me3 antibody (ab6002; Abcam) or 5 μl of EZH2 antibody (#5246, cell signaling) were used per IP. ChIP-seq libraries were prepared according to Illumina instructions and sequenced on Illumina’s Genome Analyzer IIx or HiSeq2000 instrument. Sequence reads were aligned to genomic sequence with Bowtie [47]. All mm10 genome annotations were obtained from the UCSC genome browser. HiddenDomains [48] and MACSv2 [49] identified ChIP-seq enrichment using 200 bp bins. Peak annotation was created by ChIPseeker [50] using only the peaks with posterior probabilities > 0.9. Reads at promoters, defined as a 1 kb window centered on the 5′ most TSS, were counted using htseq-count [51].

### Cell culture

Mouse embryonic fibroblasts (MEFs) were cultured as described previously [52]. Primary MEFs were synchronized by serum starvation (0.2% FBS) for 2 days and then released into cell cycle by supplement with 10% FBS. The cells were harvested at the time points indicated for RNA and protein extraction. HEK293 were cultured in the same medium as MEFs and transfected with FLAG- or HA-tagged PRC2 subunits using calcium phosphate. The cells were collected 48 h post-transfection for protein extraction. Mouse E14 ES cells were cultured in Glasgow Minimum Essential Medium supplemented with 15% fetal bovine serum, 1.0 mM l-glutamine, 0.1 mM minimal essential medium-nonessential amino acids, 0.1 mM β-mercaptoethanol, and leukemia inhibitory factor. The differentiation of ES cells with retinol acid treatment was performed as previously described [53].

### Protein extraction, histone extraction, immunoprecipitation, and Western blotting

Protein or histone was prepared from MEFs or ESCs for Western blot analysis as described [54]. For immunoprecipitation assay, 200 μg protein extract from HEK293T cells was incubated with anti-FLAG (F3165, Sigma) or anti-HA (A190-108A, Bethyl Labs) conjugated protein A/G agarose beads (sc-2003, Santa Cruz Biotechnology). FLAG- or HA-tagged full-length coding fragments of EZH1, EZH2, PHF1, PHF19, JARID2, and RBBP4 were amplified from their cDNAs and cloned into an expression vector bearing CAG promoter.

### Immunostaining on cryosections and mouse ES cells

The immunostaining on testis cryosections is as described [54]. Ezh2KO and EzhDKO ES cells were transfected with FLAG-tagged EZH variants using Xfect™ mESC Transfection Reagent (631320, Clontech) according to manufacturer’s instruction. The cells were trypsinized and resuspended in ES cell medium 48 h post-transfection. ES cells were fixed on slides by adding three volumes of 2% PFA (containing 0.1% Triton X-100 and 100 mM sucrose in 1 × PBS) at 4 °C for 30 min. The slides were air-dried and stored at 4 °C for immunofluorescence assays.

### Genome editing of *Ezh* variants by CRISPR-Cas9

sgRNAs targeting *Ezh1* and *Ezh2* were cloned into pX330-U6-Chimeric_BB-CBh-hSpCas9 (42230, Addgene) using Golden Gate assembly cloning strategy [55]. The modification of *Ezh* genes in ES cells followed the procedure as described [56]. Briefly, 5 × 10^4^ E14 ES cells were cultured on 60 mm dishes for 1 day and then transfected with plasmids expressing Cas9 and sgRNAs, along with a plasmid expressing PGK-PuroR (Addgene, Cat. No. 31937) using FuGENE HD reagent (Promega) according to the manufacturer’s instructions. The cells were treated with 2 μg/ml puromycin for 2 days and recovered in normal culture medium until ES cell colonies grew up. *Ezh1* targeted colonies were genotyped by PCR and verified by DNA sequencing. *Ezh2* targeted colonies were screened by Western blot analysis and confirmed by DNA sequencing.

## Additional files

**Additional file 1: Fig. S1.** Alternative splicing of exon 4 and exon 8 in *Ezh2* by RT-PCR analysis in testes at different ages, tissues, embryos, and cell lines.

**Additional file 2: Fig. S2.** Histone modifications, TF binding, and chromatin accessibility around the transcriptional start sites of *Ezh2*, *Eed*, and *Suz12*. (a) Histone modifications and chromatin accessibility around *Ezh2* transcriptional start sites. ChIP-seq and ATAC-seq assays were performed on spermatocytes isolated from 17-day old testes. (b) Enrichment of E2F1 at *Ezh2* promoter regions in Hela S3 cells. ChIP-seq data was retrieved from ENCODE. (c) Enrichment of H3K4me3, H3K27ac, and MYBL1 on *Eed* and *Suz12* in p17 spermatocytes by ChIP-seq analysis.

**Additional file 3: Fig. S3.** EZH variants in the restoration of H3K27 methylation in *Ezh2*KO ES cell lines. (a) Western blot analysis of H3K27 methylaiton levels in *Ezh* knockout ES cells. (b) Ectopic expression of EZH variants in *Ezh*DKO ES cells. The cells were harvested two days after transfection for Western blot analysis. (c, d) Immunofluorescence analysis of H3K27me2 and me3 in *Ezh2*KO ES cells that were transfected with FLAG-tagged EZH variants. Scale Bar: 20 μm.

**Additional file 4: Fig. S4.** EZH variants interact with other PRC2 subunits. (a) The interaction between EZH2 and EED or SUZ12 in ES cells and spermatocytes was examined by coimmunoprecipitation assays. (b, c) The interaction between EZH variants and PRC2’s accessory subunits was examined by coimmunoprecipitation assays. HEK293T cells were cotransfected with each of EZH variants and of accessory subunits and harvested for assays two days post-transfection. (d) Comparison of EZH2 protein levels between ES cells and spermatocytes by Western blot analysis. Nucleolin serves as a control.

**Additional file 5: Table S1.** Primer sequences for RT-PCR, quantitative real-time PCR, and ChIP-PCR.
