# Supplementary figures and images for: EZH2 variants differentially regulate polycomb repressive complex 2 in histone methylation and cell differentiation
Source: Epigenetics Chromatin. 2018 Dec 6;11:71. doi: 10.1186/s13072-018-0242-9 (PMC6282306; doi:10.1186/s13072-018-0242-9)

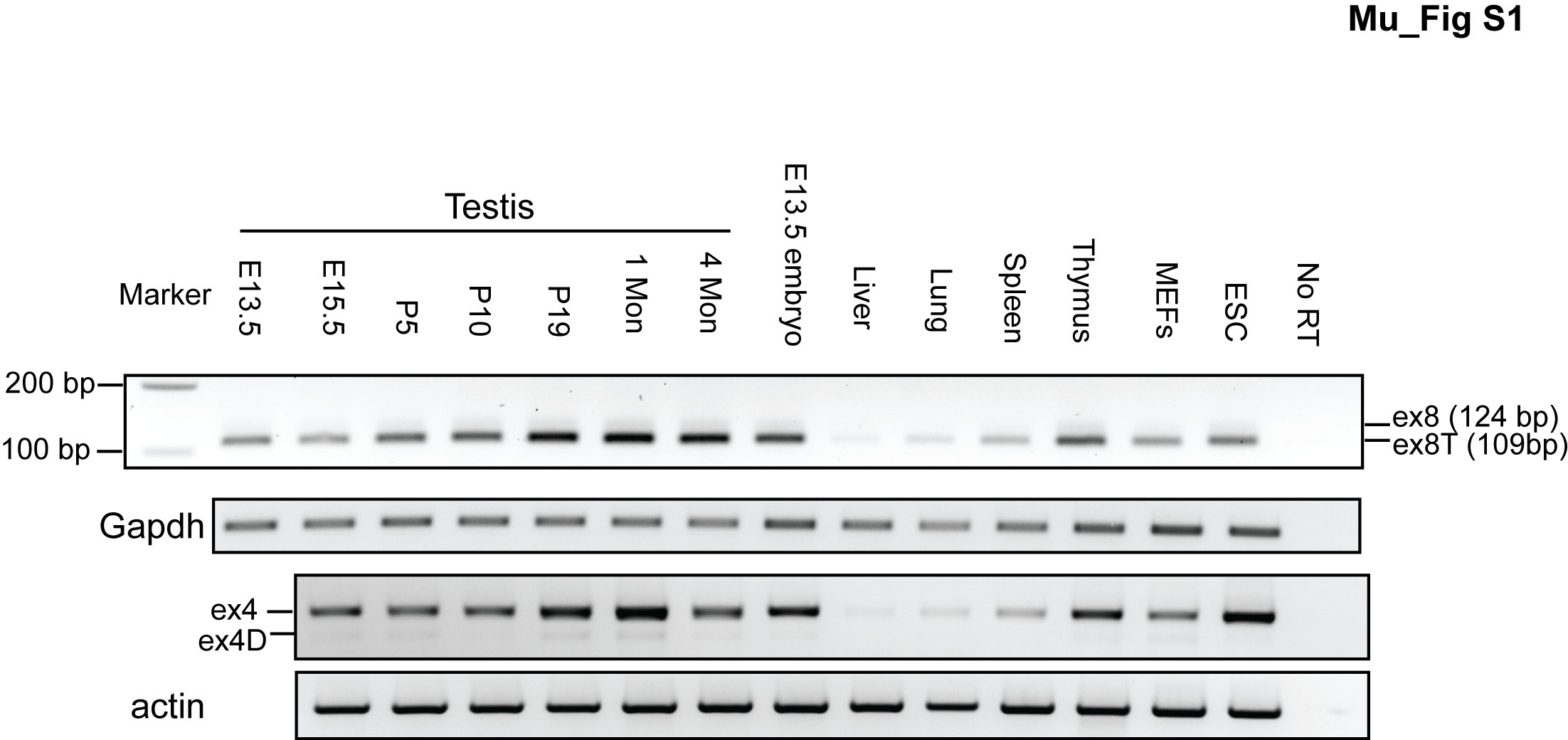

Supplement: Supplementary file 1 — Additional file 1: Fig. S1. Alternative splicing of exon 4 and exon 8 in Ezh2 by RT-PCR analysis in testes at different ages, tissues, embryos, and cell lines. [file 13072_2018_242_MOESM1_ESM.png]

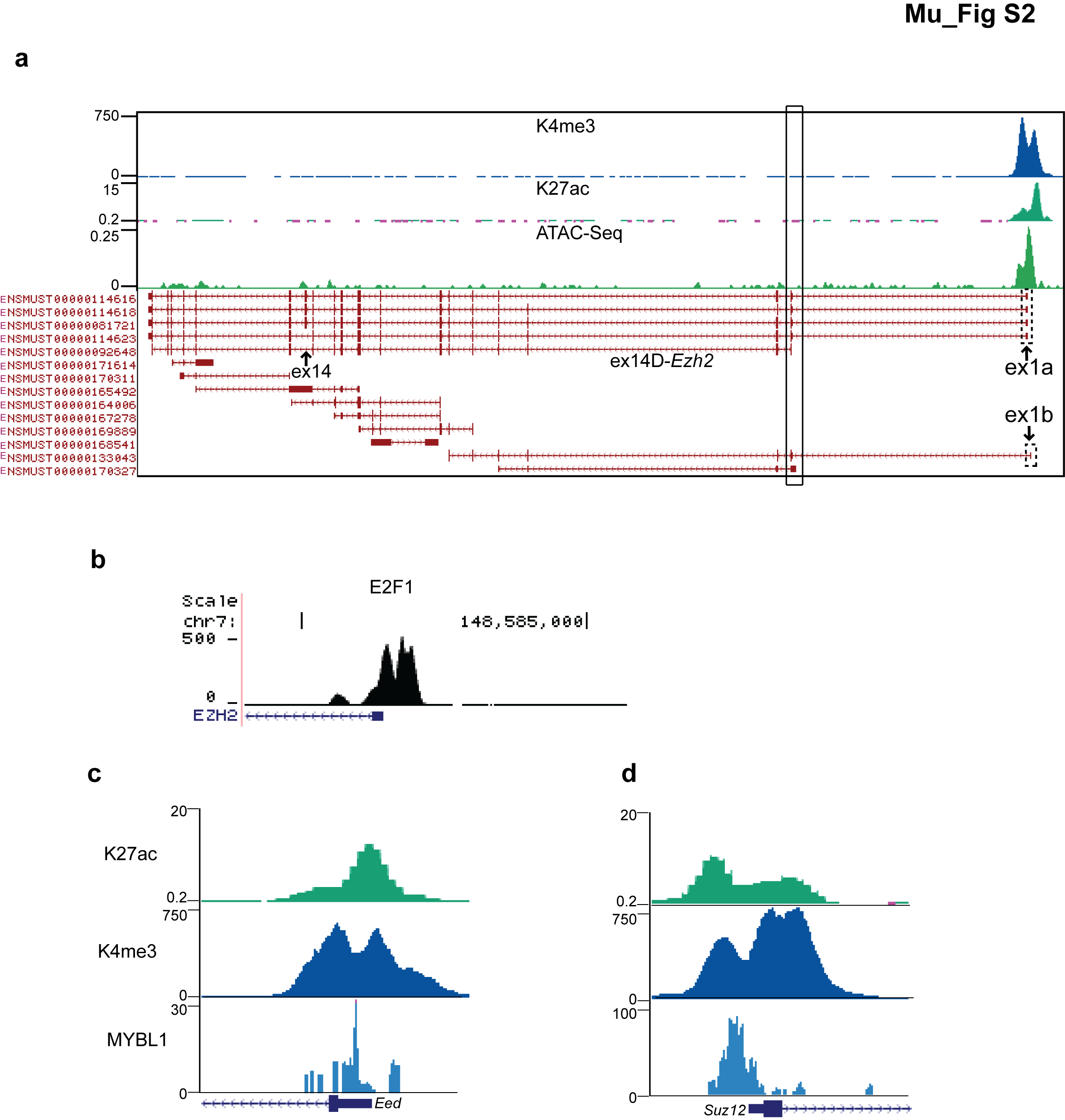

Supplement: Supplementary file 2 — Additional file 2: Fig. S2. Histone modifications, TF binding, and chromatin accessibility around the transcriptional start sites of Ezh2, Eed, and Suz12. (a) Histone modifications and chromatin accessibility around Ezh2 transcriptional start sites. ChIP-seq and ATAC-seq assays were performed on spermatocytes isolated from 17-day old testes. (b) Enrichment of E2F1 at Ezh2 promoter regions in Hela S3 cells. ChIP-seq data was retrieved from ENCODE. (c) Enrichment of H3K4me3, H3K27ac, and MYBL1 on Eed and Suz12 in p17 spermatocytes by ChIP-seq analysis. [file 13072_2018_242_MOESM2_ESM.png]

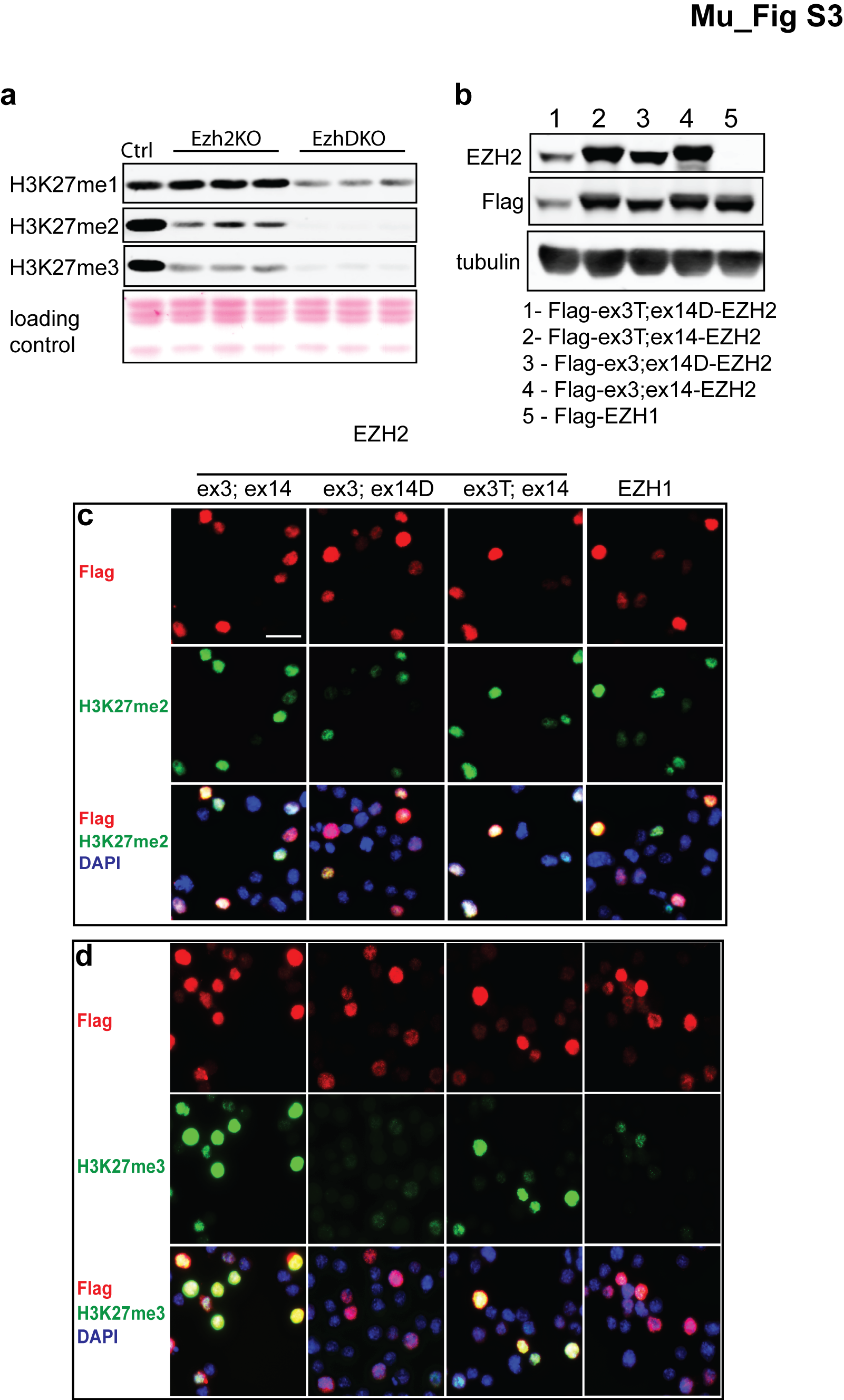

Supplement: Supplementary file 3 — Additional file 3: Fig. S3. EZH variants in the restoration of H3K27 methylation in Ezh2KO ES cell lines. (a) Western blot analysis of H3K27 methylaiton levels in Ezh knockout ES cells. (b) Ectopic expression of EZH variants in EzhDKO ES cells. The cells were harvested two days after transfection for Western blot analysis. (c, d) Immunofluorescence analysis of H3K27me2 and me3 in Ezh2KO ES cells that were transfected with FLAG-tagged EZH variants. Scale Bar: 20 μm. [file 13072_2018_242_MOESM3_ESM.png]

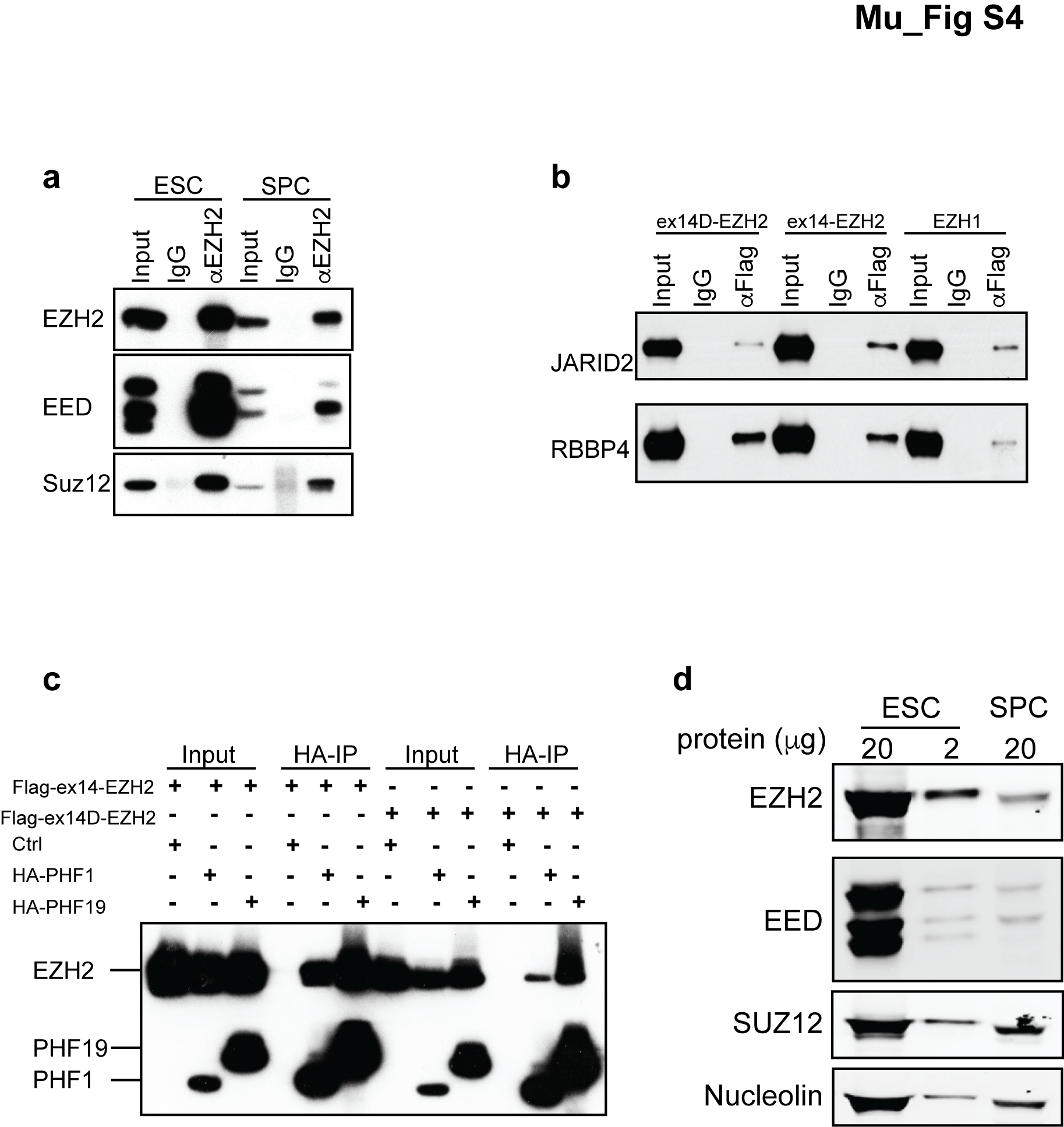

Supplement: Supplementary file 4 — Additional file 4: Fig. S4. EZH variants interact with other PRC2 subunits. (a) The interaction between EZH2 and EED or SUZ12 in ES cells and spermatocytes was examined by coimmunoprecipitation assays. (b, c) The interaction between EZH variants and PRC2’s accessory subunits was examined by coimmunoprecipitation assays. HEK293T cells were cotransfected with each of EZH variants and of accessory subunits and harvested for assays two days post-transfection. (d) Comparison of EZH2 protein levels between ES cells and spermatocytes by Western blot analysis. Nucleolin serves as a control. [file 13072_2018_242_MOESM4_ESM.png]
