# Supplementary material for: EZH2 variants differentially regulate polycomb repressive complex 2 in histone methylation and cell differentiation
Source: Epigenetics Chromatin. 2018 Dec 6;11:71. doi: 10.1186/s13072-018-0242-9 (PMC6282306; doi:10.1186/s13072-018-0242-9)
Supplement: Supplementary file 5 — Additional file 5: Table S1. Primer sequences for RT-PCR, quantitative real-time PCR, and ChIP-PCR. [file 13072_2018_242_MOESM5_ESM.docx]

**Table S1.** Primer sequences for RT-PCR, quantitative real time PCR, and ChIP-PCR

|  | Forward (5’ - 3’) | Reverse (5’ - 3’) |
| --- | --- | --- |
| **RT-PCR** |  |  |
| Ezh2ex3 | CCTTAAACCAAGAGTGGAAGCA | CAGGGTCTTTAACGGGATGA |
| Ezh2ex4 | AAGCAGCGGAGGATACAGC | CTGGATCATCTCCATCATCG |
| Ezh2ex8 | GCTCTTCTGTCGACGATGTTT | AGGCTTGTTGTCCAAAGCTG |
| Ezh2ex14 | CCTGTTCCCACTGAGGATGT | AGACGGTGCCAGCAGTAAGT |
| Ezh2-5’UTR | TGTCCATGGCTTTCCTGAGA | GAGGTCAGGGTCACACTCTC |
| Gapdh | ctggagaaacctgccaagta | tgttgctgtagccgtattca |
| actin | CGAGCGTGGCTACAGCTTCACC | CCGATCCACACAGAGTACTTGC |
| **qPCR** |  |  |
| Ezh2ex3 | ACTTCTGTGAGCTCATTGCG | GGGTCTTTAACGGGATGACTTG |
| Ezh2ex3T | GCACATCATGACTTCTTGTTCAG | CGACTGCATTCAGGGTCTTTA |
| Ezh2ex14 | CTATCAACCCTGTGACCATCC | TGACACTCTGAACTACATTGACA |
| Ezh2ex14D | GATACAACTGAAAAAGGGTCAAA | AGCCAGGTAGCATGGACACT |
| Ezh2 | CAGGATGAAGCAGACAGAAGAGGA | TCGGGTTGCATCCACCACAAA |
| Ezh1 | GTCTTCCACGGCACCTATTT | TGTTGGCAGCTTTAGGATAAGT |
| Eed | CCAAACCTTCTCCTGTCAGTAA | TCAGCACTCAGAACTTCATCTC |
| Suz12 | GGACAGGAGAAACCAACGATAA | AGCGTCTCCTTAACAGCAATAG |
| Rbbp4 | CTACACAAAGCACCCTTCTAAAC | GGATTCCAAGAAAGCCCATAAC |
| Rbbp7 | CTTTATGGGACCTGCGTAATCT | GTACCACTTGAGGCCAGAATAG |
| Brachyury | CATGCTGCAGTCCCATGATA | AGACTGGGATACTGGCTAGAG |
| Mixl1 | CATGACATCACTCACCTTTCTTTG | CTCGATGGAGATGGCTATAGTTAAT |
| Eomes | AAGAACGAGTGCCCGGTGCTATTA | AGCCCACTGTTAACTCAAGGTCCA |
| **ChIP-PCR** |  |  |
| Hoxa10 | CTGGCTCTTGAACCTGTACCCC | CAAGGGTGCTTCCAAATAGTC |
| Hoxd9 | GGATAATCGCCTAGGTGTGACTTAG | CATCTCTTCTTGCCTCTCTGGG |
| Pax7 | AGCAGAAAGAGGCGCTGAGAG | CTGAGCCCAGAGGTTGCG |
| Bmp6 | AGCCGCCTCTGAGGGTTC | GCCAGGTGTGTCCTAGGCAG |
| Elavl3 | CCTGGTTCGAGAGGCTTTG | ACAGCCTTCCAGTGAGGA |
| Shc3 | CATGAGTGCCACCAGGAAG | GTTACCCACCTTGACCACATAG |
| Runx2 | CACGACAACCGCACCAT | CACGGAGCACAGGAAGTT |
| Fgf4-Pro | GAAAGGTTCTGGCGGTTCA | CGCACTCGAACCTGTTGAT |
| Fgf4-Intr | GTATGTTCATGGCCCTCAGTAA | GTCACAGTCTAGGAAGGAAGTG |
| Utf1 | CTTGGGCGACATCTCAACA | AAACGGTTTGGTCGAAGGA |
| Nphs1 | CCAGGATGGTCAGGTTTGTT | GTGGCGAGTGTTGGAGAG |
